# Phosphorylated tau/amyloid beta 1-42 ratio in ventricular cerebrospinal fluid reflects outcome in idiopathic normal pressure hydrocephalus

**DOI:** 10.1186/2045-8118-9-7

**Published:** 2012-03-23

**Authors:** Sunil Patel, Edward B Lee, Sharon X Xie, Anica Law, Eric M Jackson, Steven E Arnold, Christopher M Clark, Leslie M Shaw, M Sean Grady, John Q Trojanowski, Roy H Hamilton

**Affiliations:** 1 Department of Neurology, University of Pennsylvania School of Medicine, Philadelphia, PA 19104, USA; 2 Institute on Aging and Center for Neurodegenerative Disease Research, University of Pennsylvania, Philadelphia, PA 19104, USA; 3 Department of Biostatistics and Epidemiology, University of Pennsylvania School of Medicine, Philadelphia, PA 19104, USA; 4 University of Pennsylvania School of Medicine, Philadelphia, PA 19104, USA; 5 Department of Neurosurgery, University of Pennsylvania, Philadelphia, PA 19104, USA; 6 Penn Memory Center, University of Pennsylvania, Philadelphia, PA 19104, USA

**Keywords:** Alzheimer's disease, Normal pressure hydrocephalus, Ventriculoperitoneal shunting, Tau, Amyloid beta 1-42, Cerebrospinal fluid

## Abstract

**Background:**

Idiopathic normal pressure hydrocephalus (iNPH) is a potentially reversible cause of dementia and gait disturbance that is typically treated by operative placement of a ventriculoperitoneal shunt. The outcome from shunting is variable, and some evidence suggests that the presence of comorbid Alzheimer's disease (AD) may impact shunt outcome. Evidence also suggests that AD biomarkers in cerebrospinal fluid (CSF) may predict the presence of AD. The aim of this study was to investigate the relationship between the phosphorylated tau/amyloid beta 1-42 (ptau/Aβ1-42) ratio in ventricular CSF and shunt outcome in patients with iNPH.

**Methods:**

We conducted a prospective trial with a cohort of 39 patients with suspected iNPH. Patients were clinically and psychometrically assessed prior to and approximately 4 months after ventriculoperitoneal shunting. Lumbar and ventricular CSF obtained intraoperatively, and tissue from intraoperative cortical biopsies were analyzed for AD biomarkers. Outcome measures included performance on clinical symptom scales, supplementary gait measures, and standard psychometric tests. We investigated relationships between the ptau/Aβ1-42 ratio in ventricular CSF and cortical AD pathology, initial clinical features, shunt outcome, and lumbar CSF ptau/Aβ1-42 ratios in the patients in our cohort.

**Results:**

We found that high ptau/Aβ1-42 ratios in ventricular CSF correlated with the presence of cortical AD pathology. At baseline, iNPH patients with ratio values most suggestive of AD presented with better gait performance but poorer cognitive performance. Patients with high ptau/Aβ1-42 ratios also showed a less robust response to shunting on both gait and cognitive measures. Finally, in a subset of 18 patients who also underwent lumbar puncture, ventricular CSF ratios were significantly correlated with lumbar CSF ratios.

**Conclusions:**

Levels of AD biomarkers in CSF correlate with the presence of cortical AD pathology and predict aspects of clinical presentation in iNPH. Moreover, preliminary evidence suggests that CSF biomarkers of AD may prove useful for stratifying shunt prognosis in patients being evaluated and treated for this condition.

## Background

Idiopathic normal pressure hydrocephalus (iNPH) is a potentially reversible cause of cognitive and motor impairment in older adults. The most widely-used and proven current treatment is placement of a ventriculoperitoneal (VP) shunt. While rates of response to treatment with VP shunts are encouraging, the factors that predict shunt unresponsiveness remain poorly understood. One potential contributor to shunt unresponsiveness is the presence of comorbid neurologic conditions that are common in aging, such as Alzheimer's disease (AD). Studies investigating the influence of comorbid AD pathology on shunt outcome have produced conflicting results. Some evidence indicates that comorbid AD contributes to a poorer response to shunting in patients with iNPH [1], while other data suggest that patients with the clinical features of iNPH benefit from shunting irrespective of the presence of AD pathology [2-4]. We recently reported data indicating that the presence of amyloid beta 1-42 (Aβ1-42) and tau--the pathologic hallmarks of AD--in cortical biopsies obtained at the time of shunt placement is associated with poorer response to shunting in patients with suspected iNPH [1].

Mounting evidence suggests that levels of amyloid beta and tau obtained from cerebrospinal fluid (CSF) samples may be used to identify patients with pathological changes indicative of AD at autopsy [5,6]. Amyloid beta 1-42 and hyperphosphorylated tau (ptau) have been more specifically implicated in the underlying pathological mechanisms of Alzheimer's disease, and the ratio of ptau/Aβ1-42 has been shown to accurately predict the presence or absence of AD [5]. Based on our prior observation of the relationship between cortical AD pathology and shunt unresponsiveness, we predicted that levels of these biomarkers in CSF may differentiate iNPH patients who respond well to shunting from those who do not.

The placement of a shunt in patients with suspected iNPH provides an opportunity to obtain and analyze ventricular CSF. We investigated the relationships between the ptau/Aβ1-42 ratio in ventricular CSF and cortical AD pathology, baseline presentation, and postsurgical outcome of patients with suspected iNPH. We predicted that there would be a strong correlation between this ratio and cortical biomarker levels. We further predicted that ratio values suggestive of higher levels of AD pathology would be associated with poorer performance on a variety of baseline and outcome measures.

## Methods

### Subjects

This study was approved by the Institutional Review Board of the University of Pennsylvania. All subjects gave informed consent prior to participation. The patients ranged in age from 64 to 85 years (mean = 75, SD = 5.8). Education ranged from 12 to 20 years (mean = 14, SD = 2.5). Patient and caregiver estimated disease duration ranged from 1 to 16 years (mean = 3, SD = 2.6). Diagnosis of iNPH required the following: 1) Presence of two of the following symptoms or signs: a) a progressive "magnetic" gait impairment, b) progressive urinary incontinence, and c) progressive cognitive impairment, and 2) ventriculomegaly on head computerized tomography (CT) scan or brain magnetic resonance imaging (MRI) out of proportion to generalized atrophy with an Evan's Index ≥ 0.3 [7]. Exclusion criteria included comorbid neurologic or psychiatric conditions and cognitive performance that was too poor to allow for assessment of meaningful change (this criterion was included in order to avoid statistical floor effects). Over a 33-month period, 78 individuals were referred for evaluation; 47 were diagnosed with iNPH and underwent surgery; 39 met both study inclusion and exclusion criteria, were enrolled and had ventricular CSF analyzed after shunting. Of the 8 that were not enrolled, 2 declined to participate in the study, 2 demonstrated poor cognitive performance, and 4 had comorbid neurologic or psychiatric conditions. Thirty of the 39 enrolled subjects followed up and were evaluated postoperatively. Of the 9 enrolled subjects who did not follow up, three experienced unrelated health complications that prohibited follow-up (one of these died), three experienced surgical complications from shunt placement that prevented outpatient evaluation (one of these died), and three declined for reasons unrelated to their health status.

### Clinical assessment

Study participants were assessed prior to surgery and approximately 4 months postoperatively (mean = 120 days; SD = 30 days) by a single behavioral neurologist. Clinical symptom severity was rated using a 15-point NPH Scale [8] consisting of three five-point subscales describing deficits in gait, cognition, and bladder control (3 = profoundly impaired, 15 = normal). Supplemental gait measures included time to walk 50 feet (in seconds), number of steps required to turn 180 degrees, and overall quality of gait as judged by the neurologist on a 5-point scale ranging from 0 (normal) to 4 (non-ambulatory) [9]. Cognitive evaluation was performed using a psychometric battery comprised of the WMS-III logical memory subtest, a 10-item word list recall task, digit span forward and reversed, category fluency, Boston Naming Test (first 30 items), trail making (A & B; total time and errors), digit symbol, and clock draw and copy. Patient performance was normalized (using Z-scores) relative to that of 202 age- and education-matched normal control subjects who participate in research at the Penn Memory Center. A global composite measure of psychometric performance was created by averaging the Z-scores. In a small number of sessions, one or more psychometric tests could not be performed due to cognitive or behavior problems, physical impediments, or verbal refusal on the part of patients.

### Shunting, tissue biopsy and analysis, and CSF acquisition and analysis

Strata II programmable valves (Medtronic, Inc., Minneapolis, MN, USA) with an initial setting of 1.5 were used in this study. All shunts were placed in the right prefrontal cortex. Prior to shunt placement, cortical tissue (approximately 0.5 cm^3^) was biopsied at the site of shunt entry. There were no surgical complications associated with biopsy acquisition. The biopsied tissue was immersion fixed in neutral buffered formalin overnight followed by paraffin embedding. Six-micrometer-thick sections were stained with hematoxylin and eosin for neuropathological examination, and with thoflavin S for identification of neuritic plaques and neurofibrillary tangles. Sections were also immunohistochemically labeled for Aβ plaques (NAB228, a mouse monoclonal antibody raised against Aβ1-11 synthetic peptide) [10], hyperphosphorylated tau for neurofibrillary tangles and dystrophic neurites (PHF1) [11], alpha-synuclein for Lewy bodies and Lewy neurites (SYN303) [12], and TDP-43 for pathological inclusions (rabbit polyclonal antibody; Protein-Tech Group, Chicago, IL, USA) with methods as previously described [13]. Control sections processed without primary antibodies were included in all runs and displayed no specific staining. Sections obtained from patients with autopsy-confirmed AD were used as positive controls. Semiquantitative scores were assigned corresponding to the densities of neurofibrillary tangles and Aβ plaques (as assessed by immunohistochemistry), and to neuritic plaques (as assessed by thioflavin S stain). Scores were as follows: absent = 0; rare = 0.5; low = 1; moderate = 2; and high = 3. Neuritic plaque scores were based on Consortium to Establish a Registry for Alzheimer's Disease criteria [14].

Ventricular CSF samples (approximately 25 cc) were obtained intraoperatively. For a subset of 18 patients, lumbar CSF was also collected by lumbar puncture (LP) performed either during the course of outpatient clinical evaluation (two patients; approximately 40 cc) or intraoperatively (16 patients; approximately 5-10 cc; after administration of anesthesia, but before shunt placement). The times between lumbar and ventricular CSF collection for the two patients who had LP during outpatient clinical evaluation were 21 and 121 days. All samples were analyzed for total tau, phosphorylated tau and beta amyloid-42 levels using the xMAP multiplex immunoassay on the Luminex platform (Luminex Corporation, Austin, TX, USA) using Innogenetics (INNO-BIA AlzBio3; Ghent, Belgium) research reagents. These reagents included monoclonal antibodies specific for Aβ1-42 (4D7A3), total tau (AT120) and ptau (AT270) [5].

### Statistical analysis

To evaluate for potential selection bias, two sample t-tests were performed to compare patients who followed up with those that did not with respect to age, education, estimated disease duration, baseline performance on clinical symptom scales, supplemental gait measures, and psychometric tests, and ventricular and lumbar CSF biomarker levels. Paired t-tests were used to determine whether the cohort of patients who completed the study improved in response to shunting. A one-sample *t*-test was used to evaluate percent change in gait time. Logistic regression was performed using forward selection with an entry *p *value of 0.25 to determine which of the three ventricular CSF biomarkers were most useful in predicting the presence of cortical pathology. Receiver operating characteristic (ROC) analysis was performed to determine the optimal ptau/Aβ1-42 cutoff for differentiating between patients with cortical AD pathology and those without. Standard decision statistics such as the area under the ROC curve (AUC), specificity, and sensitivity were generated by means of a logistic regression model [15]. In order to ensure that the effect of the ptau/Aβ1-42 ratio in predicting cortical pathology was not confounded by age, education, and disease duration, Pearson correlations were run between the ratio and these variables. Based on the cutoff level, the cohort was then divided into low (n = 17) and high (n = 22) ratio groups. For each study measure, a two sample *t*-test was performed to assess the influence of biomarker ratio on baseline performance. The influence of biomarker level on shunt response was assessed using a repeated measures ANOVA for each study measure with study measure as the dependent variable and pre/post surgical status (within subject) and ratio group (between subject) as the independent variables. A two sample *t*-test was used to determine if the percent change in gait time was dependent on biomarker levels. Finally, Pearson correlations were employed to investigate the relationship between the ventricular and lumbar levels of ptau/Aβ1-42 in the subset of patients who underwent LP. Regression parameters for predicting lumbar CSF ratio values from ventricular CSF ratio values were also determined. Statistical analysis was performed using SAS Software (Version 9.2, SAS Institute Inc., Cary, NC, USA). All statistical tests were two-sided with a 0.05 significance level unless otherwise indicated.

## Results

### Baseline characterization and shunt response of entire cohort

At baseline, the mean NPH scale total score was 9.5 (N = 39; SD = 1.7). The mean subscale scores were as follows: mean gait score = 3.2 (N = 39; SD = 0.7), mean cognition score = 3.6 (N = 39; SD = 0.6), and mean incontinence score = 2.8 (N = 38; SD = 1.1). The distribution of subscale scores is shown in Table 1. Of the 39 patients tested at baseline, 32 (82%) presented with the full triad of symptoms. The mean quality of gait scale score was 1.6 (N = 38; SD = 0.9). The mean global composite Z-score at baseline was -1.0 (N = 33; SD = 0.6).

**Table 1:** Clinical presentation of cohort at baseline

	Frequency (%)		
**Subscale Score**	**Gait**	**Cognition**	**Incontinence**

1	1 (2.56)	0 (0)	4 (10.53)

2	2 (5.13)	0 (0)	12 (31.58)

3	27 (69.23)	19 (48.72)	13 (34.21)

4	8 (20.51)	18 (46.15)	6 (15.79)

5	1 (2.56)	2 (5.13)	3 (7.89)

Clinical symptom severity was rated using a 15-point NPH scale comprised of three five-point subscales describing deficits in gait, cognition, and bladder control. Numbers in each cell indicate how many subjects in the cohort were given a specific subscale score ranging from 5 (normal) to 1 (profoundly impaired). Numbers in parentheses represent the percentage of the cohort with a given NPH subscale score.

At baseline, patients who followed up showed poorer performance on the cognition subscale of the NPH scale (t = 2.69, df = 37, *p *= 0.011) and trended toward poorer performance on the global composite Z-score (t = 1.92, df = 31, *p *= 0.064) compared to the group of patients who did not follow up (see Additional file 1 for detailed statistical results for all variables that were analyzed). No other statistically significant differences between these two groups were observed. In subsequent analyses detailed below, all 39 patients were divided into a group with a high ptau/Aβ1-42 ratio in ventricular CSF and a group with a low ptau/Aβ1-42 ratio, using a statistically determined cutoff value. Of the 9 patients who did not complete the study, 4 were in the low ratio group and 5 were in the high ratio group.

For patients who followed up, there was significant improvement between baseline and postoperative performance on the NPH scale total score (t = 6.16, df = 29, *p *< 0.001), all of its subscales (Gait t = 6.11, df = 29, *p *< 0.001; Cognition t = 3.75, df = 29, *p *= 0.001; Incontinence t = 5.07, df = 28, *p *< 0.001), quality of gait (t = 3.53, df = 29, *p *= 0.001), proportional change in gait time (t = 3.10, df = 28, *p *= 0.004), and global composite Z-score (t = 3.99, df = 20, *p *= 0.001). Of the component tests of the global composite, there was significant improvement in category fluency (t = 2.78, df = 28, *p *= 0.01) and digit symbol (t = 2.87, df = 26, *p *= 0.008) and a trend towards improvement in trails A time (t = 1.84, df = 28, *p *= 0.077). (Additional file 2 shows the statistical test results for improvement on all instruments.)

### Relationship between ventricular CSF biomarker levels and cortical AD pathology

In the 37 patients who underwent cortical biopsy, 25 (67.6%) demonstrated evidence of one or more AD pathologic markers, while 12 (32.4%) showed no evidence of AD pathology. In the 39 patients who had ventricular CSF analyzed: mean Aβ1-42 value was 121 pg/ml (SD = 36, range = 37-234); mean total tau value was 306 pg/ml (SD = 155, range = 62-861); and mean ptau value was 47 pg/ml (SD = 22, range = 10-109).

**Figure 1:**
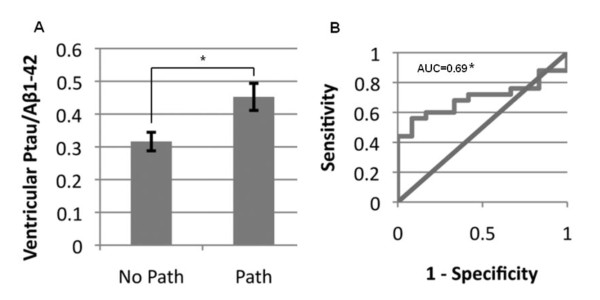
**Stratification of subjects by cortical p-tau and Aβ1-42 pathology**. A) Ventricular ptau/Aβ1-42 ratio values stratified by the presence (path) or absence (no path) of cortical pathology. B) ROC curve for differentiation of the no path and path groups by the ventricular ptau/Aβ1-42 ratio. Error bars represent standard error, * *p *< 0.05, n = 37.

Logistic regression using forward selection revealed that Aβ1-42 (beta = -0.02, point estimate = 0.98, *p *= 0.043) and ptau (beta = 0.03, point estimate = 1.03, *p *= 0.122) were the most significant CSF biomarkers for predicting the presence of cortical tissue pathology. T-tests showed that the ventricular CSF ptau/Aβ1-42 ratio values differed significantly (t = 2.73, df = 35, *p *= 0.01) between patients with cortical tissue pathology (mean = 0.45, SD = 0.21) and those with no cortical pathology (mean = 0.32, SD = 0.1) (Figure 1A). ROC analysis revealed the optimal ptau/Aβ1-42 cutoff for differentiating patients with pathology from those without pathology to be 0.37 (AUC = 0.69, *p *= 0.025) (Figure 1B). With this cutoff, sensitivity was 68% and specificity was 67%. Pearson correlation revealed no significant relationship between ventricular CSF ptau/Aβ1-42 ratio and education, age, or patient/caregiver estimated disease duration (all *p *> 0.1).

### Influence of ventricular biomarker levels on baseline performance

Patients with higher CSF ptau/Aβ1-42 ratio values showed significantly poorer baseline performance on Trails B time (t = 2.4, df = 32, *p *= 0.022) and digit symbol (t = 2.27, df = 34, *p *= 0.03). This group trended towards poorer performance on digit span backwards (t = 1.9, df = 37, *p *= 0.065) and trails B errors (t = 1.88, df = 32, *p *= 0.069). Patients with a ptau/Aβ1-42 ratio > 0.37 showed better gait performance as assessed by the quality of gait scale (t = 2.04, df = 36, *p *= 0.049) and gait subscale of the NPH scale (t = 2.97, df = 37, *p *= 0.005). (See Additional file 3 for detailed statistical results for all variables that were analyzed.)

### Influence of ventricular biomarker levels on shunt response

**Figure 2:**
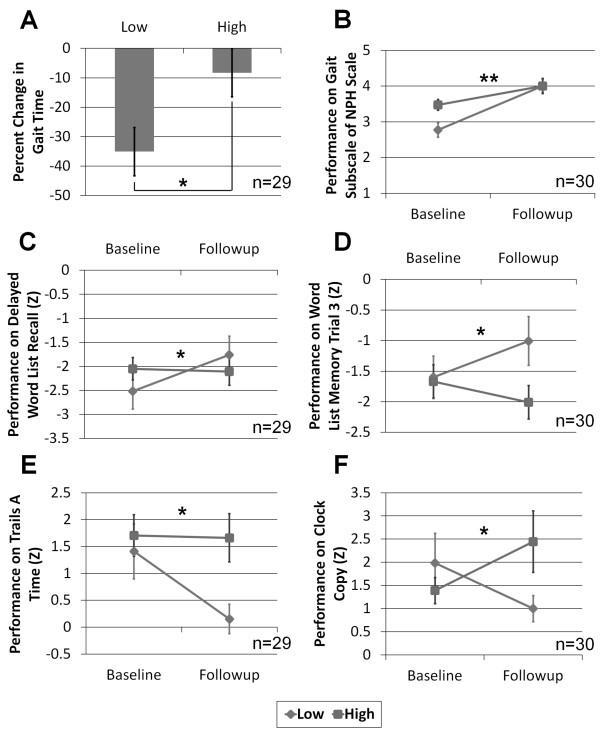
**Impact of ventricular CSF ptau/Aβ1-42 ratios on outcome measures**. Shunt outcome with respect to gait (**A**, **B**) and cognition (**C**-**F**) stratified by high and low ptau/Aβ1-42 ratio values. The high/low cutoff for the ratio was 0.37. Error bars represent standard error. * *p *< 0.05, ** *p *< 0.01.

Significant interactions between session (pre/post surgery) and ptau/Aβ1-42 ratio group were observed for the gait subscale of the NPH scale (F[1,28] = 8.09, *p *= 0.008), trails A time (F[1,27] = 4.56, *p *= 0.042), word list memory trial 3 (F[1,28] = 6.89, *p *= 0.014), word list recall (F[1,27] = 4.29, *p *= 0.048), and clock copy (F[1,28] = 4.62, *p *= 0.040); in all cases, patients with a higher ptau/Aβ1-42 ratio showed less robust response to shunting than those with a lower ratio. A *t*-test revealed a significantly (t = 2.26, df = 27, *p *= 0.032) lower percent change in gait time in the group with higher ptau/Aβ1-42 (mean = -8.3, SD = 33.4) compared to the group with lower ptau/Aβ1-42 (mean = -35.1, SD = 28.3). (See Figure 2 and Additional file 4 which shows detailed statistical results for all variables that were analyzed.)

### Relationship between biomarker levels in the ventricular and lumbar CSF spaces

The ratios of ptau/Aβ1-42 were positively correlated (N = 18, r = 0.63, *p *= 0.005) between ventricular and lumbar CSF samples. The ventricular CSF ptau/Aβ1-42 ratio significantly predicted the lumbar CSF ptau/Aβ1-42 ratio (beta = 0.22, t(16) = 3.27, *p *= 0.005; intercept = 0.03, t(16) = 0.88, *p *= 0.392): ratio_lumbar _= 0.22 × ratio_ventricular _+ 0.03.

## Discussion

We investigated relationships between the ptau/Aβ1-42 ratio in ventricular CSF and cortical AD pathology, initial clinical features, shunt outcome, and lumbar CSF ptau/Aβ1-42 ratios in patients being shunted for iNPH. We found that the ventricular CSF ptau/Aβ1-42 ratio differentiated patients with cortical AD pathology from those without pathology with a sensitivity of 68% and a specificity of 67% at the optimal cutoff point. Patients with the highest levels of ventricular ptau/Aβ1-42 (more suggestive of AD) performed more poorly at baseline on a variety of psychometric measures but performed better on gait measures. Higher ptau/Aβ1-42 ratios were associated with poorer improvement in both cognition and gait after shunting. Finally, we demonstrated a significant correlation between ptau/Aβ1-42 ratios in the ventricular and lumbar CSF in a subset of our cohort, suggesting that measures of lumbar ptau/Aβ1-42 may also correlate with patterns of baseline performance and shunt outcomes in patients with suspected iNPH.

The impact of AD pathology on the specific cognitive measures used in this study is informative. Improvement in the entire cohort of subjects after shunting was most significant on certain tests of executive function, attention, and speeded psychomotor action (category fluency, digit symbol, and trails A time), and not memory. Deficits in these cognitive domains (classically considered part of a constellation of frontostriatal or so-called "subcortical" deficits) have long been associated with iNPH [16], such that improvement in these areas in response to shunting was expected. However, it is notable that significant baseline differences were identified between subjects with high and low ptau/Aβ1-42 ratios in ventricular CSF on measures that also tested these aspects of cognition (Trails B, digit span and digit symbol). The finding that comorbid AD pathology worsens performance in these specific areas reinforces the notion that it is difficult to disambiguate patients with the clinical syndrome of iNPH from those with co-morbid AD based on patterns of cognitive impairment. On the other hand, the finding that patients with high ventricular CSF ptau/Aβ1-42 ratios have better baseline gait performance suggests that cognitive symptoms tend to predominate the overall clinical picture of those patients who are affected by comorbid AD and iNPH. The finding that patients with high ptau/Aβ1-42 ratios show less recovery of gait function after shunting is accounted for at least partly by their higher baseline gait performance compared to patients lacking comorbid AD pathology.

Patients with high ptau/Aβ1-42 ratios showed poorer post-shunt improvement mainly on tests of memory (immediate and delayed word list recall) and visuospatial function (clock copy)--cognitive domains that are typically impaired in patients with AD. In other words, while the baseline cognitive profile of these patients may not readily distinguish patients with comorbid AD pathology from those without, their response to shunting may suggest selective failure of improvement in cognitive domains typically associated with AD. Examining individual psychometric measures more closely, there also emerged an interesting dissociation between performance on different memory tests in our battery. While the ptau/Aβ1-42 ratio made a difference with respect to outcomes for verbal list learning, it did not impact logical memory performance. One possible explanation is that the two tasks differ with respect to specific cognitive demands. Evidence suggests that performance on story memory tasks relies, to some extent, on intrinsic semantic organization of the material, while word-list memory tasks require subjects to self-generate organizational strategies between unrelated items [17]. Word list memory is thus thought to rely more on executive function, a domain thought to be preferentially affected in iNPH [16]. The finding that patients with low ptau/Aβ1-42 ratios show selective improvement in word list recall is therefore potentially consistent with the idea that iNPH may influence performance on word list recall tasks to a greater extent than performance on story memory tasks. However, the finding that baseline logical memory performance did not differ significantly between high and low ptau/Aβ1-42 ratio groups was somewhat unexpected, since more severe baseline impairments in this area might have been predicted in patients with AD pathology.

Our finding that total tau does not predict the presence of cortical AD pathology in patients with iNPH is consistent with a previous study which found elevated total tau levels in the lumbar CSF of patients with iNPH and those with AD [18]. In addition, Tisell and colleagues [19] have investigated the relationship between ventricular total tau levels and shunt outcome in iNPH, reporting no significant relationship between total tau and shunt response as assessed using measures of gait, balance, alertness, and cognition. One likely explanation for the difference between these findings and our own is our use of a ratio of biomarkers that may be more specifically associated with AD pathological processes than total tau. This is further supported by a study conducted by Tarnaris and colleagues [20] that found that total tau achieved a sensitivity of only 17% and a specificity of only 20% for predicting favorable shunt outcome, as assessed by determining the extent to which patients demonstrated non-transient improvement and resumption of pre-illness activity at 6 months. Interestingly, the combination of total tau and Aβ1-42 predicted favorable outcome with a sensitivity of 80% and a specificity of 82.4%.

Ventricular CSF biomarker information, although not available as part of a typical clinical outpatient evaluation for iNPH, may potentially be used to guide postoperative management for those patients who receive magnetically programmable shunts. CSF AD biomarker data obtained intraoperatively may identify a subset of patients for whom repeated shunt adjustments are unlikely to yield clinical benefit, thus sparing those patients from additional risk of overdrainage and other complications. Bauman and colleagues [21] recently observed that cortical pathology data may similarly inform shunt management strategy. In addition, ventricular biomarker levels consistent with AD may potentially suggest that a patient previously presumed to have iNPH should be re-evaluated and possibly treated for AD.

Our finding that ptau/Aβ1-42 ratios in the ventricular and lumbar CSF are correlated suggests that lumbar ptau/Aβ1-42 may also predict baseline performance and shunt outcome in patients with suspected iNPH. The regression equation suggests that the optimal cutoff ratio for the lumbar CSF is much smaller than the ventricular CSF ratio. This is consistent with the findings of Tarnaris and colleagues [22] that the levels of various biomarkers, including total tau and Aβ1-42, differ between the ventricular and lumbar CSF. Although we have provided a regression equation that predicts the lumbar CSF ptau/Aβ1-42 ratio from the ventricular ratio, the optimal lumbar ratio cutoff should ideally be determined by replicating our analysis in a larger sample using lumbar CSF biomarkers directly.

This study has several limitations. One is its relatively small sample size. In addition, a number of patients who had received shunts to treat presumed iNPH did not participate in the study, either because they were excluded by the study criteria or because they failed to follow up. Restriction of the patients participating in the study in these ways may limit the generalizability of the findings and may also have led to a degree of selection bias. A comparison of baseline performance between patients who completed the study versus those who enrolled but did not complete the study reveals a statistically significant difference in the cognitive subscale measure of the NPH scale. Notably, this result was not supported by performance on any individual psychometric instrument. Nonetheless, this finding suggests that patients who completed the study may have had slightly worse baseline cognition than those who dropped out. While we believe that the results of the study are still valid and informative, we also acknowledge that the data may be somewhat more informative for patients who have cognitive impairments as part of their presenting clinical picture. Finally, we assessed outcome at 4 months following surgery. A longer timeframe for assessment of outcome in these patients may be more useful for gauging long-term outcome and we acknowledge the possibility that clinical and cognitive characteristics of these patients may evolve long after 4 months following surgery. However, given that the temporal dynamics of post-surgical symptom improvement in iNPH patients are not well characterized, these findings do contribute meaningfully to what is currently known about recovery of function after shunting.

## Conclusions

To date, the underlying pathology and pathophysiology of iNPH remain largely unknown. While the results of this study are consistent with the notion that CSF ptau and Aβ1-42 are markers of co-morbid AD, it is also possible that these indicators may, for some patients, represent part of the pathological profile of iNPH as well. Nevertheless, the results of this preliminary study strongly suggest that their presence or absence in ventricular CSF correlates with certain outcomes for patients undergoing shunting. If our findings can be replicated in larger samples, they would indicate that ventricular CSF measures of ptau and Aβ1-42, and possibly lumbar measures of these biomarkers, are potentially clinically useful in predicting outcomes for patients being evaluated for iNPH.

## Competing interests

Leslie Shaw holds stock options in Saladex Biomedical and has received support from Pfizer for lecturing at ICAD 2009 and for travel to the meeting. Chris Clark is a member of a safety monitoring board for an AD clinical trial at Elan-Wyeth. He is also a member of a diagnostic adjudication board for an MCI clinical trial at Bristol Myers Squib. He has received travel support for meetings. Eddie Lee is an inventor on a patent for an Aβ antibody. This antibody was not used in the present study.

## Authors' contributions

SP performed statistical analyses and drafted the manuscript. EL conducted immunohistochemical staining of cortical biopsies. SX performed and oversaw statistical analyses. AL assisted with statistical analysis and drafting of the manuscript. EJ participated in the design of the study. SA performed semi-quantitative scoring of cortical biopsies. CC participated in the design of the study and the pre- and post-operative evaluation of subjects. LS analyzed CSF for levels of AD biomarkers. MSG participated in the design of the study, performed surgeries, and acquired CSF samples and cortical biopsies. JT contributed to the design of the study and oversaw analysis of cortical biopsies and CSF samples. RH participated in the design of the study, performed pre- and post-operative evaluation of subjects, assisted with statistical analyses, and oversaw drafting of manuscript. All authors read and approved the final manuscript.

## Supplementary Material

Additional file 1 **Assessment of potential selection bias**. Analysis of baseline measurements of patients lost to follow up compared with those evaluated both pre and postoperatively. T-tests were performed to test for baseline differences between the groups. Click here for file

Additional file 2 **Shunt response of entire cohort evaluated both pre- and post operatively**. Results of paired t-tests performed to test for improvement of entire cohort in response to shunting. Note that percent change in gait time was analyzed using a one-sample *t*-test. Click here for file

Additional file 3 **Relationship between ventricular CSF ptau/Aβ1-42 ratio and baseline presentation**. Results of t-tests performed to test for baseline differences between the low and high ventricular CSF ptau/Aβ1-42 ratio groups. The cutoff value defining the low and high ratio groups was 0.37. Click here for file

Additional file 4 **Relationship between ventricular CSF ptau/Aβ1-42 ratio and shunt response**. Results of repeated measures ANOVAs performed to test for differences in shunt response between the low and high ventricular CSF ptau/Aβ1-42 ratio groups. Only the results for the interaction between session (pre/post surgery) and ptau/Aβ1-42 ratio group are shown. Note that percent change in gait time was compared between the two groups using a *t*-test. Click here for file
